# Expect the unexpected: Ablation of an atypically located atrial tachycardia

**DOI:** 10.1016/j.hrcr.2024.05.024

**Published:** 2024-06-05

**Authors:** Cas Teunissen, Moniek G. Cox, Wil Kassenberg, Peter Loh

**Affiliations:** ∗ Department of Cardiology, Heart Lung Center Utrecht, University Medical Center, Utrecht, The Netherlands; † Department of Cardiology, University of Groningen, University Medical Centre, Groningen, The Netherlands

**Keywords:** Atrial tachycardia, Radiofrequency catheter ablation, Right atrial appendage, Supraventricular tachycardia, Angiography

**Key Teaching Points**

• Expect the unexpected and rely on the electrical signals: When no local signals are identified look for unusual origins of atrial tachycardia (AT).
• Just like the left atrial appendage, the right atrial appendage (RAA) may have various morphological variations that can act arrhythmogenically.
• Angiography of the right atrium and RAA might be helpful in case of misunderstood AT or inappropriate sinus tachycardia.

## Introduction

Focal atrial tachycardia (AT) is a relatively uncommon cause of supraventricular tachycardia. The response to antiarrhythmic drugs is often poor, but good long-term success can be achieved with radiofrequency ablation.^1^ ATs originate in both the right and left atrium. Common locations include the crista terminalis, near the tricuspid and mitral annulus, the coronary sinus, within the pulmonary veins, and at the para-Hisian area.^2^, ^3^, ^4^, ^5^ Less common origins are the atrial appendages.^6^^,^^7^ We report a case of AT originating from an anatomical variation of the right atrial appendage (RAA).

## Case report

**Figure 1:**
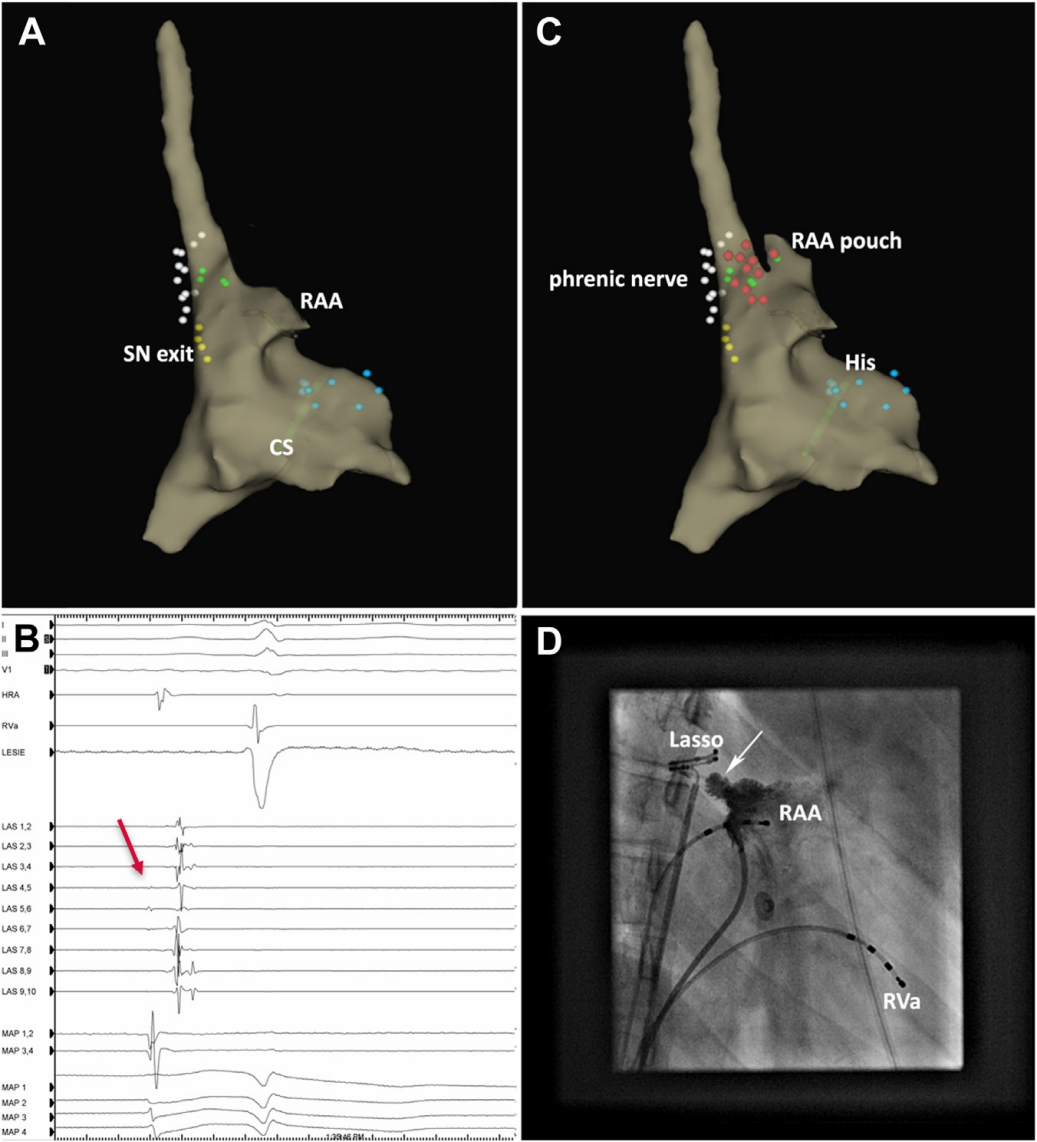
**A:** Three-dimensional reconstruction of the right atrium. Yellow dots indicate the sinus node exit during sinus rhythm, white dots the course of phrenic nerve, blue dots the bundle of His, and green dots the earliest activation recorded on the lasso catheter during tachycardia. **B:** Cardiac tracing. Early activation with remote signals detected on the circular catheter (*red arrow*) in the superior vena cava ostium. On the ablation catheter positioned in the right atrial appendage pouch, local signals with a comparable timing to the far-field signals can be appreciated. **C:** Three-dimensional reconstruction of the right atrium after the pouch of right atrial appendage is identified. Red dots indicate sites of ablation. Ablation inside the pouch terminated the atrial tachycardia. **D:** Angiography in right anterior oblique view reveals the right atrial appendage pouch (*white arrow*). The lasso catheter is positioned in the superior vena cava. CS = coronary sinus; His = bundle of His; HRA = high right atrium; LAS = decapolar circular catheter; MAP = ablation catheter; RAA = right atrial appendage; RVa = right ventricular apex; SN = sinus node.

**Figure 2:**
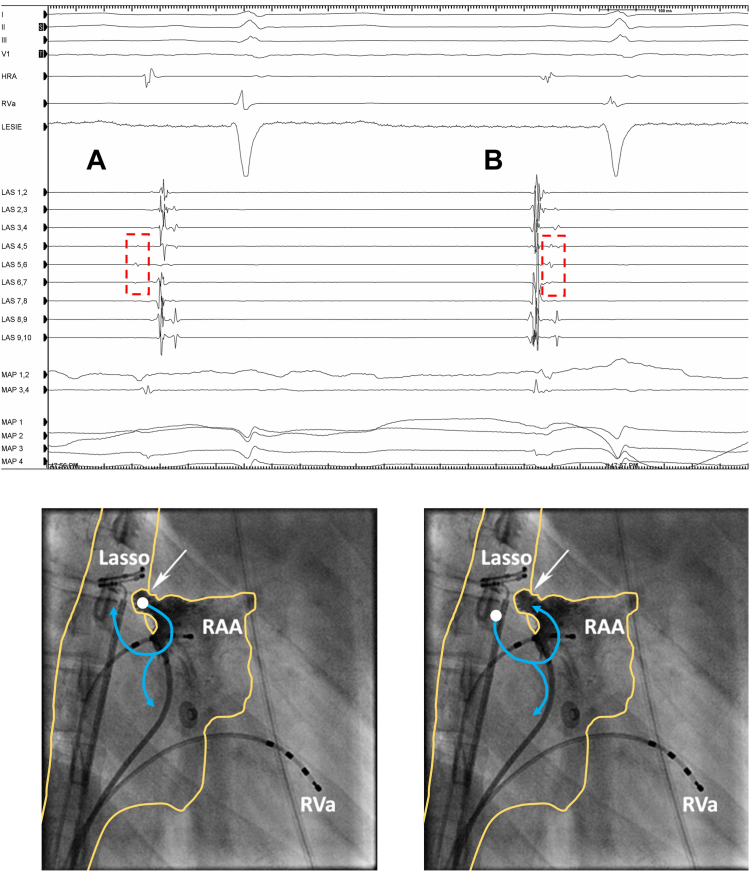
Cardiac tracing (upper panel) showing termination of atrial tachycardia (AT) during ablation with conversion to sinus rhythm and matching visual explanation (lower panel) of the difference in activation sequence of the last AT complex (**A**) and the first sinus rhythm complex (**B**). As appreciated, during AT the far-field signals on the circular catheter (*dashed square*) are the earliest detected signals, indicating that the origin of AT is in the right atrial appendage (RAA) pouch. During sinus rhythm, the RAA pouch is activated later, as shown in panel B. Upper panel: HRA = high right atrium; LAS = decapolar circular catheter; MAP = ablation catheter; RVa = right ventricular apex. Lower panel. Lasso = decapolar circular catheter in the superior vena cava; RAA = diagnostic catheter in the right atrial appendage; RVa = diagnostic catheter placed in the right ventricle.

A 17-year-old woman with a history of atrioventricular nodal reentrant tachycardia (AVNRT) ablation presented with recurrent symptoms of palpitations with sudden onset and offset, and fatigue. Twenty-four-hour Holter recording revealed a mean heart rate of 96 beats/min and 47% tachycardia. During tachycardia, P-wave morphology was comparable to sinus rhythm. Although the heart rate was initially well managed with ivabradine, symptoms did not subside. Inappropriate sinus tachycardia was suspected and electrophysiology study was performed. After 3-dimensional reconstruction (EnSite NavX; Abbott, Abbott Park, IL) of the right atrium, the exit of the sinus node was mapped (Figure 1A). Programmed electrical stimulation revealed dual atrioventricular nodal physiology, but no recurrence of AVNRT. Subsequent isoproterenol infusion spontaneously induced AT with a cycle length of 370 ms. Earliest activation was found in the anterior aspect of the superior vena cava (SVC) ostium. The right-sided phrenic nerve was mapped. For better identification of the AT origin, a decapolar circular catheter was introduced. Early signals on the circular catheter appeared far-field (Figure 1B) and ablation at the site of the earliest signals did not terminate the tachycardia. An epicardial focus was supposed but was considered highly unlikely for this location. Electrical stimulation in the RAA revealed similar far-field potentials in the SVC, followed by sharp local activation. This raised suspicion of the presence of a structure between the RAA and SVC. Mapping in the RAA revealed a cranially protruding pouch (Figure 1C) facing the SVC. The presence of this pouch was confirmed by angiography (Figure 1D). Inside this pouch, early local potentials were encountered (Figure 2A). Nonirrigated ablation with 30 W terminated AT after 5 seconds and was continued for 90 seconds (Figure 2). During a 30-minute observation period, neither AT nor AVNRT could be induced, with and without isoproterenol provocation. No recurrences occurred after 1 year of follow-up.

## Discussion

We present a case of AT originating from an anatomical variation of the RAA, which was successfully treated by radiofrequency catheter ablation. The RAA is an uncommon site for AT.^6^, ^7^, ^8^, ^9^ RAA AT tends to be incessant and may be misdiagnosed as inappropriate sinus tachycardia.^6^^,^^9^^,^^10^ Ablation therapy is often succesful.^9^

Various morphological variations of the RAA have been described, including different shapes, lobes, and aneurysms.^8^ AT can originate from both the base and the distal part of the RAA.^9^^,^^11^ Also, ATs from a distal RAA aneurysm have been described previously.^12^ AT from a cranially protruding pouch has not been described before in an adult.

## Conclusion

The RAA may have arrhythmogenic anatomical variations. Radiofrequency ablation can be a successful treatment of RAA AT.

## Disclosures

None.
